# Construction and validation of a predictive in-hospital mortality nomogram in patients with staphylococcus aureus bloodstream infection

**DOI:** 10.1038/s41598-025-15826-8

**Published:** 2025-08-13

**Authors:** Xiangquan Xie, Chuncai Wu, Jing Zhou, Shaohong Jiang, Baoying Shen, Qiaoli Xu, Chuanbin Huang

**Affiliations:** 1 Department of Infection Control, Zhangzhou Affiliated Hospital of Fujian Medical University, Zhangzhou, Fujian China; 2 The Laboratory Department, Zhangzhou Affiliated Hospital of Fujian Medical University, Zhangzhou, Fujian China; 3 Department of Critical Care Medicine, Zhangzhou Affiliated Hospital of Fujian Medical University, Zhangzhou, Fujian China

**Keywords:** Staphylococcus aureus, Bloodstream infection, Nomogram, ECFC score, Predictive model, Outcomes research, Infectious-disease epidemiology, Bacterial infection, Infection

## Abstract

We aimed to construct and validate a predictive nomogram to evaluate in-hospital mortality of patients with S.aureus BSI. A 10-year retrospective cohort design was conducted to analyze data from 484 patients diagnosed with S. aureus BSI between 2014 and 2023. Clinical data from 339 patients (2014 to 2021) were harnessed in training cohort to develop a predictive nomogram, which underwent rigorous internal validation. An independent cohort of 145 patients (2022 to 2023) were collected for external validation. The prognostic performance of the model was comprehensively assessed using AUC, calibration curve, and DCA. We ultimately identified several key factors that were incorporated into the final prognostic nomogram: the ECFC score, the CCI score, procalcitonin levels, admission to the intensive care unit, and multimicrobial BSI. Internal validation was assessed via 5-fold cross-validation, repeated 400 times on the training cohort, yielding an average AUC value of 0.930 vs. 0.940 of the total. External validation further confirmed the nomogram’s accuracy, with an AUC value of 0.929. Additionally, the calibration curves and DCAs revealed excellent consistency and substantial net clinical benefits in both cohorts. The development of this predictive nomogram marks a substantial breakthrough in the management of patients with S. aureus BSI.

## Introduction

‌Staphylococcus aureus (S. aureus) is a highly prevalent and virulent pathogen that can cause a diverse array of infections, from mild skin infections to life-threatening conditions such as sepsis and endocarditis^1–4^. According to data from the China Antimicrobial Resistance Surveillance System^5^*S. aureus* was the most frequently isolated gram-positive bacterium from 2014 to 2019, with its percentage progressively increasing from 8.7 to 9.6%. Bloodstream infections (BSIs) caused by *S. aureus* constitute a major global healthcare challenge and contribute significantly to patient morbidity, mortality, and healthcare costs^6–8^. ‌Furthermore, the advent of methicillin-resistant *S. aureus* (MRSA) BSI has significantly heightened the complexity and unpredictability of treatment outcomes^9,10^.

Recently, growing interest exists in developing prognostic models that can accurately predict the outcomes of patients with BSI or other infectious diseases^11–15^. These models are designed to identify early warning signs and risk factors that may affect clinical course and treatment response, thus allowing clinicians to assess disease severity more accurately and tailor treatment strategies with greater precision.

One of the cutting-edge parameters explored in BSI prognostic models is the Early Clinical Failure Criteria (ECFC) score, which was first proposed by Rac et al.^16^ showing an ECFC score between 72 and 96 h following the onset of gram-negative bacterial BSI was utilized as a comprehensive scale for predicting unfavorable outcomes. Similar to the q-Pitt score^17–19^ the ECFC score is an scoring system involving five vital signs: heart rate, systolic blood pressure, respiratory rate, consciousness, and peripheral white blood cell (WBC) count, which can be easily obtained before empirical antibiotic therapy in patients with BSI. Currently, no studies on the ECFC score for predicting the prognosis of gram-positive bacterial BSI, much less of *S. aureus* BSI.

In addition to ECFC score, numerous biomarkers and clinical factors have been proposed as potential predictors of prognosis in patients with *S. aureus* BSI, including inflammatory factors such as procalcitonin (PCT), and patient-specific factors such as age, Charlson Comorbidity Index (CCI ) score, as well as immune status among others^20,21^. Furthermore, the presence of hospital-associated BSI (HABSI)^22^ and MRSA BSI^23^both of which are associated with higher mortality and treatment failure rates, are critical considerations in predicting patient outcomes.

The study aimed to construct a predictive nomogram based on prognostic predictors for patients with *S. aureus* BSI, particularly focusing on the role of the ECFC score as a potential predictor, thereby improving the management and prognosis of patients with *S. aureus* BSI.

## Methods

### Study design and settings

This 10-year retrospective cohort study was conducted at Zhangzhou Affiliated Hospital of Fujian Medical University, a 4,060-bed tertiary general hospital in Zhangzhou, Fujian Province, China. Patients aged 18 years or older, diagnosed with *S. aureus* BSI, and hospitalized between 2014 and 2023 were eligible for inclusion. Only the first episode for each patient was included in the analysis. To construct a predictive nomogram for hospital mortality, we first divided the patient data into two cohorts according to the patient admission time. Patients hospitalized between 2014 and 2021 were included in the training cohort, while those hospitalized between 2022 and 2023 were included in the external validation cohort. A predictive model was constructed, and a nomogram was plotted according to the training cohort. Internal validation was conducted using the K-fold cross-validation technique^24^ and external validation was conducted using regression equations constructed for the training cohort^25^.

### Data collection

**Fig. 1:**
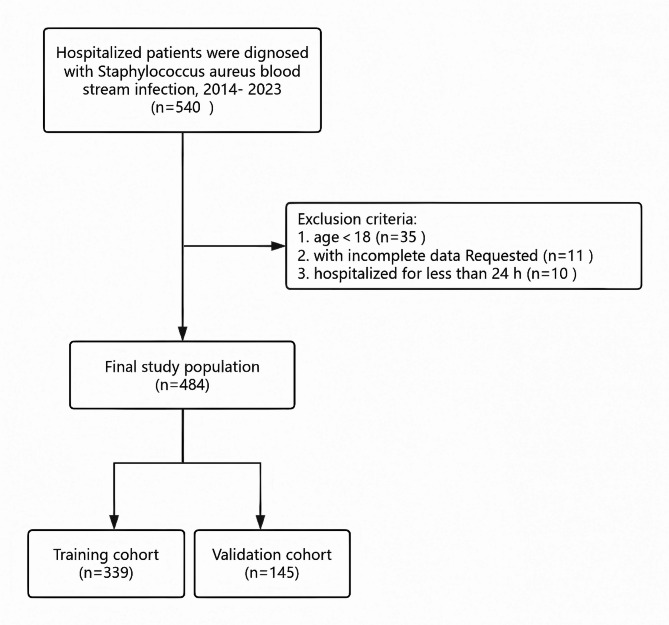
A flow chart of patient selection.

Patients with *S. aureus* BSI who were under 18 years of age, with incomplete data, as well as required hospitalized for less than 24 h, were excluded. A flow chart of patients excluded based on these criteria is shown in Fig. 1, with 11 patients that had more than 20% missing data been excluded from the analysis. The remaining dataset was processed using the “mice” package in R software to impute the missing values.

All clinical data were collected from the Hospital Information System (ZOE SOFT, Xiamen, Fujian, China), Laboratory Information System (XINGHE SOFTWARE, Shanghai, China), and the Healthcare Associated Infection Monitoring System (XINGLIN TECHNOLOGY, Hangzhou, Zhejiang, China).

The collected information included: (1) Demographics: age, sex, ICU admission, and primary infection sites, which provided a foundation for assessing the impact of infection. (2) Vital signs: temperature, heart rate, systolic blood pressure, respiratory rate, and mental status at the onset of BSI, indicating instant physical condition at the time of *S. aureus* BSI. (3) Comorbidities: Immunosuppression, malignant cancer, tumor metastasis, renal failure, diabetic complications, congestive heart failure, severe liver disease, diabetes, cerebrovascular disease, chronic pulmonary disease, and hypoproteinemia. (4) Biomarkers strongly related to BSI: WBC count, neutrophil ratio, procalcitonin. (5) Disease severity scores: ECFC and CCI scores, with the ECFC scoring criteria listed in Table 1. (6) Treatments: empirical antibiotic use, targeted antibiotic use, combined antibiotic use, and hemodialysis. (7) Outcome: All-cause in-hospital mortality.

**Table 1:** Early clinical failure criteria.

Score	Vital signs
1	•Systolic blood pressure < 100 mmHg or vasopressor use
1	•Heart rate > 100 beats/minute
1	•Respiratory rate ≥ 22 breaths/minute or mechanical ventilation
1	•Altered mental status
1	•Peripheral white blood cell count > 12 000/mm^3^

### Definitions

An *S. aureus* strain showing resistance to methicillin in the antimicrobial susceptibility test is defined as MRSA according to Clinical and Laboratory Standards Institute M100^26^ and a case diagnosed with BSI 48 h after admission is regarded as HABSI of *S. aureus*. In addition, primary *S. aureus* BSI means no other sites of *S. aureus* infection occurred before the onset of the BSI.

### Statistical analysis

SPSS software (version 19.0 (IBM Corporation, Armonk, NY, USA)), R Language software (version 4.2.3, R Core Team, Vienna, Austria), and Zstats v1.0 (www.zstats.net) were used for the statistical analyses. All tests were two-tailed. Categorical variables are expressed as absolute frequencies or percentages, and Chi-square or Fisher’s exact tests were used for statistical comparisons. Quantitative variables are expressed as mean ± standard deviation for normal distribution or median + range for skewed distribution and were compared using Student’s t-test or the Mann-Whitney U-test. To avoid multicollinearity and overfitting, a least absolute shrinkage and selection operator (LASSO) regression analysis^27^ was initially used to filter the variables, and factors with non-zero coefficients were selected for further analysis with multivariate logistic regression (MLR) analysis, and a nomogram model was established using predictors with *P* < 0.1 in regression analysis.

Furthermore, we evaluated the predictive performance of the nomogram via the area under the curve (AUC) of the receiver operating characteristic (ROC) curve in the training cohort. Internal validation was conducted using 5-fold cross-validation and external validation was conducted in the time-period validation cohort using the same equation as in the training cohort. Calibration curves were used to measure the consistency between the predicted probabilities and observed outcomes. The Decision curve analysis (DCA) was used to measure the clinical utility of the nomogram by calculating the net benefits of different threshold probabilities. A *P* value < 0.05 was considered statistical significance.

### Ethics approval and consent statement

This study was approved by the Ethics Committee of Zhangzhou Affiliated Hospital of Fujian Medical University (No. 2024LWB386). All methods were performed in accordance with the relevant guidelines and regulations laid down in the 1964 Declaration of Helsinki and its later amendments. Due to retrospective nature of the study, informed consent was waived by the Ethics Committee of Zhangzhou Affiliated Hospital of Fujian Medical University.

## Results

### Basic clinical characteristics

Our study included 484 patients with community-onset and hospital-associated *S. aureus* BSI (332 vs. 152, respectively). The median age of participants was 61 years old, and 316 of which (65.29%) were male. Overall, 114 (23.55%) patients experienced unfavorable outcomes, although over 98% (478/484) of the patients received empirical antibiotics and over 95% (463/484) received targeted antibiotics. As allocated by patient admission time, 339 patients ultimately accessed in the training cohort, and 145 accessed in the validation cohort. In comparison, the median ECFC score + range was 1.0 ( 0.0–2.0) in both cohorts. The median CCI scores + ranges were 3.0 (2.0–4.0) and 4.0 (3.0–5.0) in the training and validation cohorts, respectively. In the training cohort, MRSA and HABSI were found in 28.61% and 33.33% of the cases, respectively, compared to 20.0% and 26.90% in the validation cohort. Intensive care unit (ICU) admission was observed in 27.14% of cases in the training cohort and 33.10% of cases in the validation cohort. The remaining characteristics of the two cohorts are exhibited in Table 2.

**Table 2:** Demographic and clinical characteristics of participants.

Variables	Total cohort	Training cohort	Validation cohort
	*n* = 484	*n* = 339	*n* = 145
Age (years) M (Q₁, Q₃)	61 (49, 71)	58 (47, 68)	67 (55, 76)
Sex, n(%)			
Male	316 (65.29)	215 (63.42)	101 (69.66)
Female	168 (34.71)	124 (36.58)	44 (30.34)
Temperature (°C), Mean ± SD	38.2 ± 1.2	38.4 ± 1.1	37.8 ± 1.3
WBC(K/uL), M (Q₁, Q₃)	12.04 (7.81, 17.01)	12.01 (7.62, 17.35)	12.16 (8.02, 16.28)
Neutrophil(%), M (Q₁, Q₃)	86.50 (77.10, 90.62)	86.50 (77.25, 90.75)	85.60 (76.20, 90.50)
PCT(ng/mL), M (Q₁, Q₃)	1.87 (0.42, 11.41)	1.59 (0.35, 9.05)	2.19 (0.52, 14.02)
Accompanying infection			
Abdominal infection, n(%)	32 (6.61)	19 (5.60)	13 (8.97)
Lower respiratory infection, n(%)	244 (50.41)	152 (44.84)	92 (63.45)
Urinary tract infection, n(%)	38 (7.85)	24 (7.08)	14 (9.66)
Skin and soft tissue infection, n(%)	97 (20.04)	70 (20.65)	27 (18.62)
Intracranial infection, n(%)	9 (1.86)	7 (2.06)	2 (1.38)
Amount of accompanying infection, M (Q₁, Q₃)	1.0 (0.0, 1.0)	1.0 (0.0, 1.0)	1.0 (0.0, 1.0)
Empirical antibiotics, n(%)	478 (98.76)	336 (99.12)	142 (97.93)
Targeted antibiotics, n(%)	463 (95.66)	325 (95.87)	138 (95.17)
Combined antibiotics, n(%)	298 (61.57)	207 (61.06)	91 (62.76)
Respiratory rate ≥ 22 breaths/minute or mechanical ventilation, n(%)	104 (21.49)	65 (19.17)	39 (26.90)
SBP < 100 mmHg or vasopressor use, n(%)	57 (11.78)	40 (11.80)	17 (11.72)
Heart rate > 100 beats/minute, n(%)	153 (36.61)	99 (29.90)	54 (37.24)
Peripheral WBC Count > 12 000/mm^3^, n(%)	292 (60.33)	209 (61.65)	83 (57.24)
Altered mental status, n(%)	79 (16.32)	53 (15.63)	26 (17.93)
Malignant tumor, n(%)	90 (18.60)	74 (21.83)	16 (11.03)
Hemodialysis, n(%)	98 (20.25)	71 (20.94)	27 (18.62)
Immunodepression, n(%)	73 (15.08)	60 (17.70)	13 (8.97)
Hypoalbuminemia, n(%)	212 (43.80)	133 (39.23)	79 (54.48)
MRSA, n(%)	126 (26.03)	97 (28.61)	29 (20.00)
HABSI, n(%)	152 (31.40)	113 (33.33)	39 (26.90)
CRBSI, n(%)	84 (17.36)	73 (21.53)	11 (7.59)
Multimicrobial BSI, n(%)	27 (5.58)	18 (5.31)	9 (6.21)
Secondary HAI, n(%)	44 (9.09)	36 (10.62)	8 (5.52)
Primary BSI, n(%)	354 (73.14)	249 (73.45)	105 (72.41)
ICU admission, n(%)	140 (28.93)	92 (27.14)	48 (33.10)
CCI score, M (Q₁, Q₃)	3.0 (2.0, 5.0)	3.0 (2.0, 4.0)	4.0 (3.0, 5.0)
ECFC score, M (Q₁, Q₃)	1.0 (0.0, 2.0)	1.0 (0.0, 2.0)	1.0 (0.0, 2.0)
In-hospital mortality, n(%)	114 (23.55)	69 (20.35)	45 (31.03)

Abbreviations: SD, standard deviation; M, median; Q₁, 1st Quartile; Q₃, 3st Quartile; WBC, white blood cell; PCT, procalcitonin; SBP, systolic blood pressure; MRSA, methicillin-resistant staphylococcus aureus; HABSI, hospital-associated blood stream infection; CRBSI, catheter-related blood stream infection; HAI, hospital-associated infection; ICU, intensive care unit; CCI, Charlson comorbidity index; ECFC, Early clinical failure criteria.

### Filtering of predictors

**Fig. 2:**
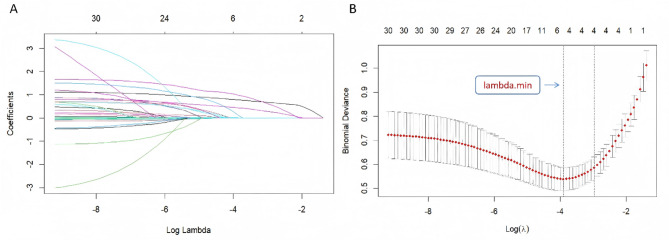
Screening of variables based on Lasso regression. **(A)** The variation characteristics of the coefficient of variables; **(B)** the selection process of the optimum value of the parameter λ in the Lasso regression model by cross-validation method.

LASSO regression analysis was conducted to preliminarily filter predictors using the predictive variables in the training cohort. The results shown in Fig. 2 indicate that five optimum variables with nonzero coefficients were filtered out when the lambda value with the smallest deviation (lambda. min), including multimicrobial BSI, PCT levels, ICU admission, the CCI score, and the ECFC score. All five factors were subsequently included in a MLR analysis using a stepwise algorithm to select the final predictive variables for model construction.

### Nomogram model construction

**Fig. 3:**
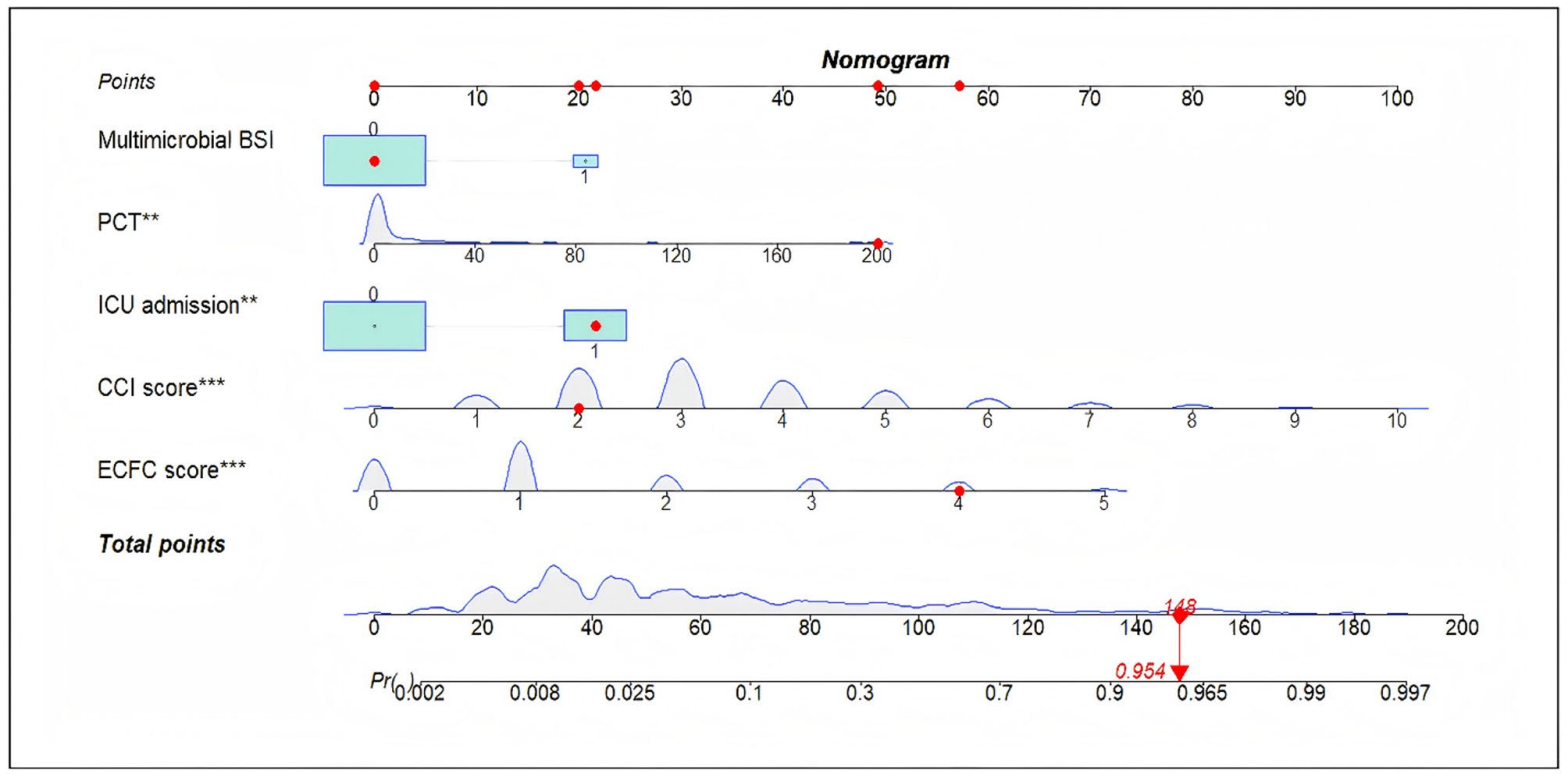
Nomogram model predicts in-hospital mortality in patients with *S.aureus* BSI. ** represents *P* value < 0.01, and *** represents *P* value < 0.001. The red dots on the nomogram represent the scores for a patient with *S. aureus* BSI, whose total score was 148, corresponding to a 95.4% probability of in-hospital mortality.

As showed in Table 3, all variables exhibited statistical significance, except for multibacterial BSI, with a *P* value of 0.090. An in-hospital mortality nomogram was plotted using all five variables (Fig. 3). The red dots on the nomogram represent the scores for a patient with *S. aureus* BSI who did not have multimicrobial BSI, was admitted to the ICU admission, got a PCT level of 200ng/ml, a CCI score of 2 and an ECFC score of 4. The patient’s total score was 148, corresponding to a 95.4% probability of in-hospital mortality. Furthermore, we built an online dynamic nomogram (URL https://xiexiangquan219628.shinyapps.io/dynamicnomogram/) for clinically application. The probability of uncomfortable prognosis and 95% confidence interval (CI) can be obtained with a simple mouse click or sliding the scale.

**Table 3:** Multivariate logistic regression analysis of predictors associated with *S.aureus* BSI.

Variables	β	S.E.	Z	*P*	OR (95%CI)
ECFC score	0.948	0.179	5.282	< 0.001	2.581 (1.815–3.669)
CCI score	0.664	0.133	4.989	< 0.001	1.942 (1.496–2.521)
ICU admission	1.433	0.452	3.168	0.002	4.192 (1.727–10.174)
PCT	0.016	0.005	2.981	0.003	1.016 (1.006–1.027)
Multibacterial BSI	1.370	0.808	1.695	0.090	3.934 (0.808–19.168)

Abbreviations: OR, Odds Ratio; CI, Confidence Interval; PCT, Procalcitonin; CCI, Charlson Comorbidity Index; ECFC, Early clinical failure criteria.

### Nomogram validation and predictive value evaluation

**Fig. 4:**
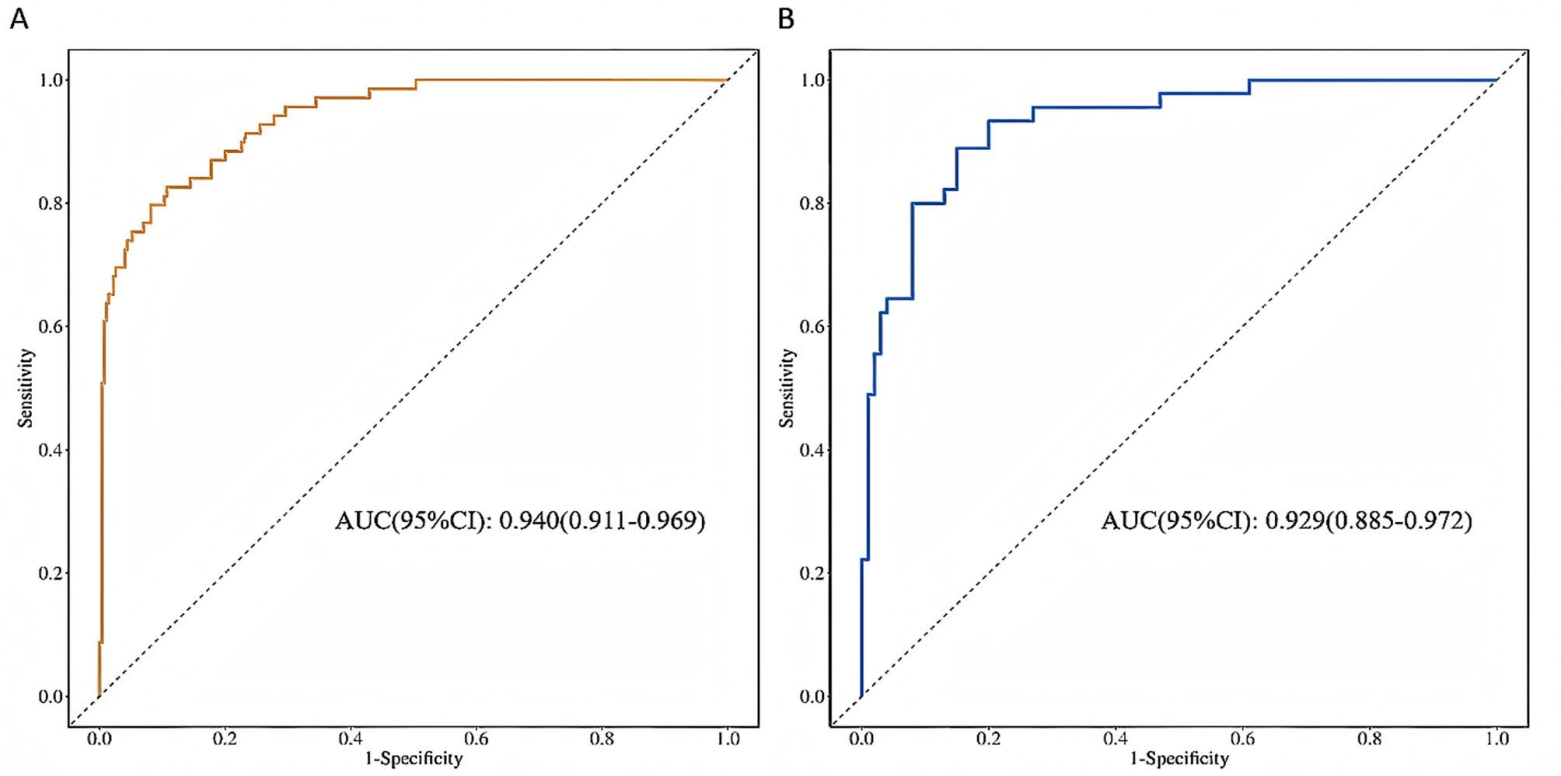
Receiver operating characteristic (ROC) curves for the nomogram with area under the curve (AUC). **(A)** Training cohort; **(B)** validation cohort.

**Fig. 5:**
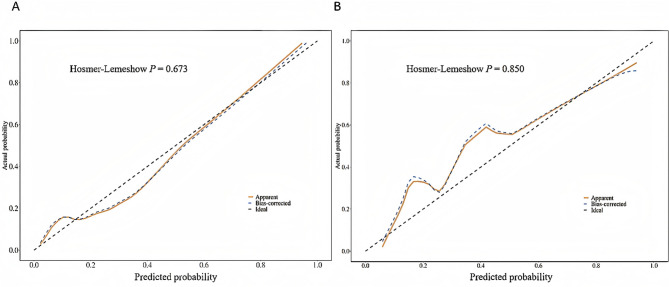
Calibration curves for the nomogram with Hosmer-Lemeshow (H-L) test. **(A)** Training cohort; **(B)** validation cohort. The X-axis shows the predicted probability of in-hospital mortality, and the Y-axis shows the observed outcomes.H-L tests show good consistency in both two cohorts, with p values of 0.673 and 0.850, respectively.

**Fig. 6:**
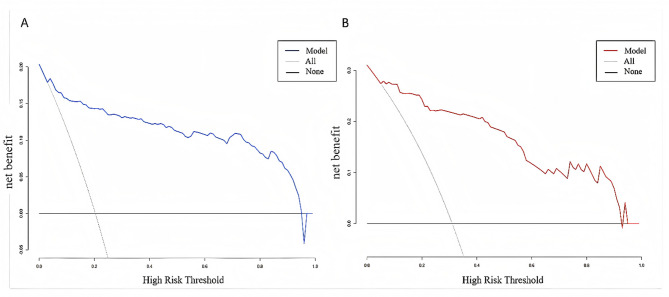
Decision curve analysis (DCA) for the nomogram. (A) Training cohort; (B) validation cohort. The X-axis indicates the threshold probability referring to the relative harm and benefit of the proposed intervention; the Y-axis indicates the standardized net benefits. Both two cohorts have positive benefits between the thresholds of 0.05 and 0.95.

A 5-fold internal cross-validation was conducted 400 times in the training cohort and revealed an average AUC value of 0.930 ± 0.035. ROC curves for the training and time-period validation cohorts are shown in Fig. 4, with AUC values of 0.940 (95% CI 0.911–0.968) and 0.929 (95% CI 0.885–0.972), respectively, indicating the outstanding accuracy of the model. Calibration curves for both cohorts are shown in Fig. 5. The results of the Hosmer-Lemeshow (H-L) tests revealed no statistical significance, with *P* values of 0.673 and 0.850, respectively. This indicates that the predicted probabilities are consistent with the actual probabilities, which enhances the reliability of the model. DCA for the nomogram is presented in Fig. 6, which demonstrates net clinical benefits when the thresholds were between 0.05 and 0.95 in both cohorts, underscoring the wide clinical practicability of the model.

## Discussion

In this study, we developed an *S. aureus* BSI prognostic prediction nomogram using a series of data collection and statistical analyses. The initial step involved partitioning the database into training and validation sets. The conventional partitioning approach typically relies on randomization^12–15^ we violated the routine and divided the database into training and time-period validation cohorts according to patient admission time (2014 to 2021 vs. 2022 to 2023). This method aligns with a large-sample study^25^ enhancing the prediction ability of the nomogram. In addition, the nomogram underwent rigorous validation using multiple methods, including k-fold cross-validation, ROC curve^28^ H-L test^29^ and DCA^30^. Each validation approach revealed excellent performance, confirming the nomogram’s robustness.

The nomogram identified several significant predictors, including the ECFC score, the CCI score, PCT levels, multimicrobial BSI, and ICU admission. These findings provide important insights into the complexities of managing *S. aureus* BSI and highlight the potential of the ECFC score as a valuable predictive tool in clinical practice.

The ECFC score, proposed by Rac et al.^16^ integrates vital signs, including respiratory rate, heart rate, blood pressure, consciousness, and peripheral WBC count, providing a comprehensive measure of a patient’s physiological status. Notably, this is similar to the q-Pitt score, although the criteria were modified by excluding the parameters of cardiac arrest and adding the peripheral WBC count, which made the score more accessible in clinical practice, thus enhancing its feasibility in clinical settings. As revealed in multivariate logistic regression, the ECFC score exhibited an odd ratio (OR) of 2.58 (95% CI 1.82–3.67), indicating that the risk of mortality in patients with *S. aureus* BSI increased by 2.58 times with every extra point in the score. The timing for determining the ECFC score suggested by Rac et al. fell within the 72-to-96-hour window following the submission of blood cultures for analysis. However, reassessment of patients’ vital signs within this timeframe may be considerably affected by the administration of empirical antibiotics and symptomatic treatments. In our study, the vast majority of patients (98.76%) were prescribed empirical antibiotics, and none were undertreated. Consequently, we determined the onset of BSI as the optimum time for assessing the ECFC score.

The inclusion of the CCI score^31^ in our model reaffirmed the established understanding that comorbidities significantly affect patient prognosis. Patients with higher comorbidity burdens often experience more severe infections and complications, underscoring the importance of comprehensive patient assessments in clinical decision-making. This finding aligns with the existing literature^32,33^ suggesting that underlying health status is a determinant prognostic factor in infectious diseases.

PCT has been increasingly recognized as a biomarker for diagnosing bacterial infections, including BSI^34,35^. However, few studies have focused on its potential prognosis. A 7-year retrospective cohort study revealed that PCT at baseline was significantly associated with 50-day in-hospital mortality among patients with community-acquired BSI^36^ which corroborated our findings that elevated PCT levels associated with poor outcomes. This suggests that PCT can serve not only as a diagnostic tool but also as a prognostic indicator, potentially guiding therapeutic decisions and resource allocation in the management of *S. aureus* BSI.

The need for ICU admission has emerged as a critical factor that reflects the severity of BSI. Patients requiring intensive care often present with more complex clinical conditions that contribute to unfavorable outcomes. This finding aligns with the existing literature that emphasizes the significance of critical care needs in the prognosis of infectious diseases^37^.

The presence of multimicrobial BSIs was also recruited as a predictor by LASSO regression, although no statistical significance was revealed in the final regression equation (*P* = 0.090). This suggests that patients with infections caused by multiple bacterial species may face increased complexity in clinical management, which can negatively affect their outcomes. These findings highlight the importance of identifying the microbiological profile of BSIs early in the treatment process to tailor appropriate therapeutic strategies.

Interestingly, our study found that several factors, such as age, immunosuppression, MRSA, HABSI and others, which have traditionally been thought to influence outcomes in previous studies^33,38,39^ did not meet the criteria for significance in our predictive model. This discrepancy may reflect the nuanced nature of *S. aureus* infections, where the presence of these factors did not independently affect outcomes when adjusted for other clinical variables. For instance, the lack of significance for MRSA infection may indicate that, while MRSA is a known pathogen associated with severe outcomes, MRSA presence alone may not sufficiently predict prognosis when other vital clinical parameters are considered. In addition, while numerous studies have explored MRSA as a prognostic factor for BSI^22,23,39^ no relevant prognostic models have been developed, leaving this association unclear. Our findings may prompt a re-evaluation of how MRSA infections are assessed in prognostic models, emphasizing the need for a multifactorial approach to patient evaluation.

There were several limitations in our study. The first limitation was that it was a single-center study, despite the large sample size. Future research should focus on validating our findings in larger and more diverse populations. The second limitation was the time-based approach to training-validation splitting (pre- and post-2021). While practical, this method could lead to confounding due to treatment evolution over time. Moreover, our study focused on *S. aureus* BSI, which may limit the generalizability of our findings to other types of bacterial infections. Additional research is needed to explore the prognostic value of the ECFC score in different infectious contexts. Despite these limitations, our study provides valuable insights into the complex interplay of clinical factors that influence outcomes in *S. aureus* BSI. By identifying key predictors of poor prognosis, we have taken a significant step toward improving patient care and outcomes in this critical area of infectious diseases.

## Conclusion

We constructed a prognostic prediction nomogram for *S. aureus* BSI, which highlighted the importance of the ECFC score, CCI score, PCT levels, ICU admission, and multimicrobial BSI as significant predictors of clinical outcomes. This model provides a practical tool for clinicians to assess risk and guide management strategies more effectively.

## Data Availability

The datasets used and/or analysed during the current study are available from the corresponding author on reasonable request.
